# Dynamin-Catalyzed Membrane Fission Requires Coordinated GTP Hydrolysis

**DOI:** 10.1371/journal.pone.0055691

**Published:** 2013-01-31

**Authors:** Ya-Wen Liu, Juha-Pekka Mattila, Sandra L. Schmid

**Affiliations:** Department of Cell Biology, The Scripps Research Institute, La Jolla, California, United States of America; University of Birmingham, United Kingdom

## Abstract

Dynamin is the most-studied membrane fission machinery and has served as a paradigm for studies of other fission GTPases; however, several critical questions regarding its function remain unresolved. In particular, because most dynamin GTPase domain mutants studied to date equally impair both basal and assembly-stimulated GTPase activities, it has been difficult to distinguish their respective roles in clathrin-mediated endocytosis (CME) or in dynamin catalyzed membrane fission. Here we compared a new dynamin mutant, Q40E, which is selectively impaired in assembly-stimulated GTPase activity with S45N, a GTP-binding mutant equally defective in both basal and assembly-stimulated GTPase activities. Both mutants potently inhibit CME and effectively recruit other endocytic accessory proteins to stalled coated pits. However, the Q40E mutant blocks at a later step than S45N, providing additional evidence that GTP binding and/or basal GTPase activities of dynamin are required throughout clathrin coated pit maturation. Importantly, using *in vitro* assays for assembly-stimulated GTPase activity and membrane fission, we find that the latter is much more potently inhibited by both dominant-negative mutants than the former. These studies establish that efficient fission from supported bilayers with excess membrane reservoir (SUPER) templates requires coordinated GTP hydrolysis across two rungs of an assembled dynamin collar.

## Introduction

Dynamin is a large, self-assembling GTPase required for clathrin-mediated endocytosis (CME). Dynamin has an atypically low affinity for GTP (10–100 µM), and a robust basal rate for GTP hydrolysis (∼0.4–1 min^−1^) that can be stimulated >100-fold by self-assembly into spiral-like structures, either in solution at low salt or on membrane templates [1]–[3]. Dynamin is recruited at low levels to nascent clathrin coated pits (CCPs) [4]–[6] and has been proposed to regulate early stages of CME [7]–[9]. An additional burst of recruitment is observed at later stages of CME [4]–[6], which presumably reflects the self-assembly of dynamin into collar-like structures at the necks of deeply-invaginated CCPs to mediate membrane fission and clathrin coated vesicle release. Functional analysis of dynamin mutations has provided significant mechanistic insight into its role in CME (reviewed by [10]–[12]). These studies have revealed that dynamin-mediated membrane fission requires GTP binding and hydrolysis [13]–[15], GTP-driven conformational changes [15], [16], membrane binding [17]–[19], self-assembly [20], [21] and curvature generation through shallow insertions into the lipid bilayer [22]. Although dynamin has been extensively studied over the past 20 years, there remains considerable controversy and uncertainty as to its exact role(s) in CME and the mechanism of dynamin-catalyzed membrane fission [8], [10], [23].

Recently, dynamin-catalyzed membrane fission has been reconstituted using supported bilayers with excess membrane reservoir (SUPER) templates as substrate [24]. There is a direct correlation between the degree to which various dynamin mutants inhibit CME *in vivo* and their ability to catalyze membrane fission and vesicle release from these templates [24], [25]. These data demonstrate both that vesicle formation from SUPER templates faithfully reconstitutes dynamin activity *in vitro* and that the major function of dynamin *in vivo* is to catalyze membrane fission. In addition to a direct role for membrane fission, we and others have suggested that dynamin also functions at early stages to monitor and/or regulate the rate of CCP maturation [4], [6], [7], [26]. This function is thought to require dynamin's basal GTP binding and hydrolysis activities.

Structural studies have shown that the C terminal helix of the distal GTPase effector domain (C_GED_) of dynamin docks onto the N- and C-terminal helices of the GTPase (G)- domain [27], [28]. Activation of the basal GTPase activity of a minimal G domain-C_GED_ fusion protein (referred to as GG) requires dimerization and structural studies have revealed conserved residues in the composite catalytic center formed by G domain dimers required for efficient GTPase activity [16]. This dimerization occurs across sequential rungs of a dynamin helix [29] and accounts for dynamin's assembly-stimulated GTPase activity. Despite the identification of mutations that selectively inhibit assembly-stimulated, but not basal, GTPase activities, there exists some uncertainty as to both the magnitude of dynamin's basal GTPase activity [15], and whether it also requires G domain dimerization (see for example [10]). Moreover, the role(s) for dynamin's GTPase activities in CME have been difficult to resolve because the G domain mutations studied thus far equally inhibit both and also greatly reduce the affinity of dynamin for nucleotide [15], [16], [30]. Another unanswered question is what is the minimum functional unit required for dynamin-catalyzed fission, is it a single ring or a dynamin spiral, and if the latter how long? While dynamin mutants defective in self-assembly are unable to catalyze membrane fission *in vitro* [24] or support CME *in vivo* [20], [21], a recent study has suggested that dynamin scaffolds might negatively regulate fission [31]. To resolve these issues, we have analyzed, *in vivo* and *in vitro*, the activities of the Q40E dynamin-1 mutant, previously reported to selectively impair assembly-stimulated GTPase activity [16], in comparison to the well-characterized S45N mutant that is defective in both GTP binding and hydrolysis.

## Results

### Biochemical properties of Q40E

Stimulated GTP hydrolysis requires dimerization of the dynamin G domains [16]. In this configuration, the amino acid D180 functions *in trans* through a series of hydrogen bonds to orient Q40 and S41 in the active site for more efficient catalysis of GTP hydrolysis. Earlier studies showed that the assembly-stimulated GTPase activity of the S41N mutant was partially impaired, while the Q40E and D180N mutants were severely impaired in assembly-stimulated GTPase activity. When measured at 500 µM GTP, the basal GTPase activity of Q40E and S41N were unaffected, while for as yet unexplained reasons, D180N exhibited ∼10-fold higher basal activity [16]. Based on these results we have chosen to further characterize Q40E, as a potentially new class of dynamin mutants selectively defective in assembly-stimulated GTPase activity.

**Figure 1:**
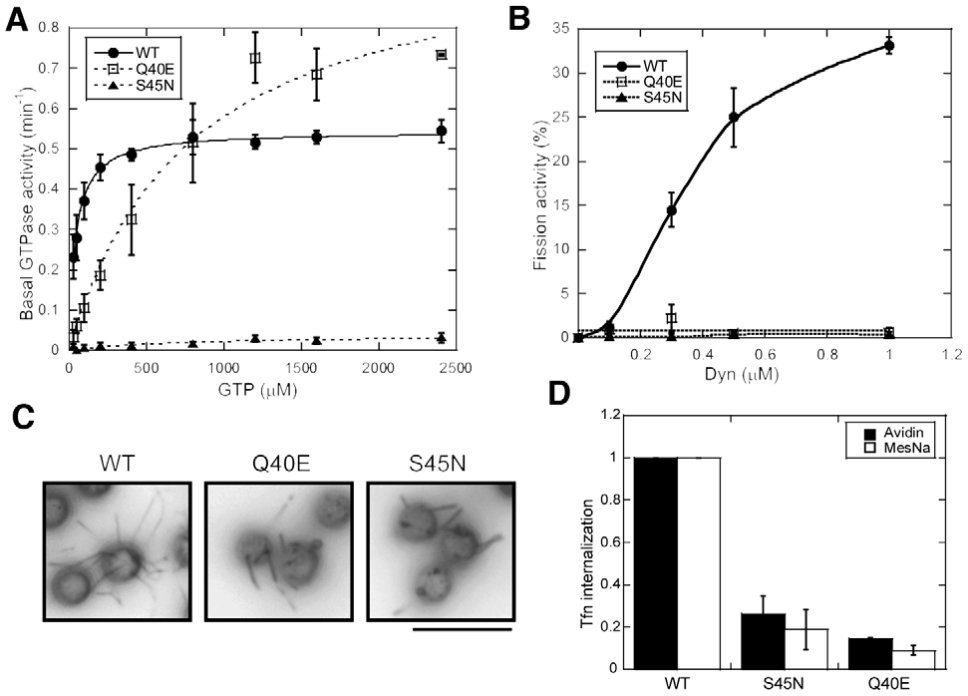
Biochemical properties of Q40E and S45N dynamin mutants. (A) Steady-state basal GTP hydrolysis of 0.5 µM WT or mutant dynamin-1 was measured at 37°C with different concentrations of GTP. Results shown are the specific GTPase activity for each condition, and data are average ± std. dev. from 3 independent experiments. (B) Membrane fission activity of the indicated concentrations of WT or mutant dynamin-1 was determined by its ability to release vesicles from SUPER templates. Fission activity is expressed as a fraction of the total membrane released from the SUPER templates during a 30 min incubation at room temperature, as described in *Experimental Procedures*. (C) The tubulation of SUPER templates by 1 µM WT or mutant dynamin-1 after incubation for 10 min at room temperature in the absence of GTP was determined by fluorescence microscopy. Images are inverted in contrast for clarity. Scale bar, 10 µm. (D) Dominant-negative effect on clathrin-mediated endocytosis. tTA-HeLa cells acutely expressing the indicated dynamins were incubated with BSSTfn for 3 min at 37°C. The efficiency of BSSTfn internalization was determined by its inaccessibility to quenching reagent, either avidin or MesNa, and normalized to dynamin-1 WT expressing cells. Data are average ± std. dev. from 3 independent experiments.

We first measured basal and assembly-stimulated GTPase activities of full length WT, S45N and Q40E dynamin-1 over a range of GTP concentrations. We confirmed the strong inhibition of assembly-stimulated GTPase activity over all GTP concentrations (data not shown, but see [16]) and that at 500 µM GTP the basal GTPase activity of Q40E was comparable to WT (Figure 1A). Indeed, basal GTPase activity does not require Q40, as mutation of this residue in the context of the minimal GTPase-GED fusion protein (GG) has no effect on its basal GTPase activity (Figure S1 in File S1). These data establish that basal GTPase activity occurs independently of the dimerization mechanism required for assembly-stimulated GTPase activity.

Unexpectedly, the rightward shift in the curve for Q40E relative to WT indicated a significant decrease in GTP binding affinity (Figure 1A). Because the basal rate of GTP hydrolysis is slow (∼1 min^−1^) relative to the rate of GTP dissociation (∼1.6 s^−1^) [32], the Km for basal GTPase activity approximates the Kd for GTP binding (see Methods), which is difficult to measure directly given the low affinity of dynamin for GTP [33]. The apparent affinity of Q40E for GTP was >10-fold reduced relative to WT dynamin-1 (Table 1). Thus, Q40E impairs GTP binding as well as GTP hydrolysis. As expected, mutation of the highly conserved active site serine to asparagine (S45N) abrogates GTP binding and hydrolysis due to steric hindrance of the bulky Asn side-chain in the active site (Figure 1A, Table 1).

**Table 1:** Michaelis-Menten constants for WT and mutant dynamin-1.

	*K_m_* (µM)	*k_cat_* (min^−1^)
WT	40±16	0.71±0.27
Q40E	755±57	0.99±0.30
S45N	>2000	<0.07±0.01

Although impaired at low concentrations of GTP, at [GTP] >500 µM, the basal activity of Q40E is comparable to WT dynamin-1. Therefore we next examined the ability of Q40E to mediate membrane fission in the presence of 1 mM GTP. Under these conditions, Q40E is as defective in catalyzing membrane fission as S45N (Figure 1B). These data directly establish that dynamin's basal GTPase activity alone is insufficient to drive membrane fission and that assembly-stimulated GTPase activity is essential for dynamin-catalyzed vesicle release from SUPER templates. The inability of these mutants to drive membrane fission is not due to a defect in membrane binding, self-assembly or curvature generation, as Q40E and S45N were indistinguishable from WT in their abilities to generate tubules from SUPER templates in the absence of GTP (Figure 1C).

### Effects of dynamin mutants in cultured cells

The intracellular [GTP] ranges from 100–1000 µM, with the average concentration in cultured tumor cells reported to be 473±214 µM [34]. At 450 µM GTP, the ratio of Dyn·GTP/Dyn( = [GTP]/K_m_) for WT is ∼11, but only ∼0.6 for Q40E. Thus, at steady state, ∼90% of WT will be in the GTP-bound form, whereas only ∼40% of Q40E will be GTP-bound. Under these conditions, the basal rate of GTP hydrolysis by Q40E will be ∼80% of WT (Figure 1A). Thus, although significantly impaired in its ability to bind GTP relative to WT, under *in vivo* conditions the Q40E mutant nonetheless retains significant basal GTP binding and hydrolysis activities relative to S45N. Therefore, we wondered whether we could detect any differential effects of overexpression of these dominant-negative mutants on the intermediates that accumulated during clathrin-mediated endocytosis.

Mutations defective in GTP binding and basal GTP hydrolysis, such as S45N or K44A, have previously been shown to block CME at intermediate stages of CCV formation [35]. In contrast, we would predict that the Q40E mutant, which is severely defective in membrane fission (Figure 1B), fully able to self-assemble (Figure 1C), and only partially impaired in basal GTPase activity under physiological conditions (Figure 1A) might function to facilitate basal GTPase-dependent early stages in CCP maturation and accumulate as collars at the necks of constricted CCPs. The accumulation of these late-stage intermediates can be detected using transferrin biotinylated via a disulfide linkage (BSSTfn) as a ligand to follow CME, based on its relative accessibility to extracellular avidin and MesNa [36] [36] [36] [36] [36]. BSSTfn that is sequestered in constricted CCPs becomes inaccessible to avidin, but remains accessible to the small membrane impermeant reducing agent, MesNa [9]. One caveat to this analysis, however, is that the BSSTfn is also bulky and would not be expected to penetrate constricted CCPs that accumulated during the prolonged 16 hr expression of mutant dynamins. Indeed, we did not detect a difference in the extent of inhibition of BSSTfn uptake using avidin or MesNa assays in cells measured 16 h after infection with adenoviruses encoding the mutant dynamins (unpublished data). Therefore, in an attempt to identify this intermediate, we induced acute protein expression. For this purpose, cells were infected with tet-regulatable (tet-off system) adenoviral expression vectors encoding WT or mutant dynamins in the presence of tetracycline for 2 hours after which tetracycline was washed away and cycloheximide was added to allow mRNA to accumulate without protein production for 3 hours. Acute protein expression was induced by removal of cycloheximide. The expression of exogenous dynamin-1 reached ∼20-fold over endogenous total dynamin within 1 hour (Figure S2A in File S1). Both S45N and Q40E dominant-negative mutants severely impaired BSSTfn internalization; however, even under these conditions we could not detect a difference in inhibition using avidin or MesNa (Figure 1D). As CCPs assemble and mature in ∼1 min [4], [5], [37], our inability to detect the accumulation of constricted CCPs could still be due to the inability of the bulky BSSTfn ligand to access receptors trapped in these intermediates. Therefore, we looked for morphological differences in the structure of CCP intermediates, first by fluorescence microscopy.

**Figure 2:**
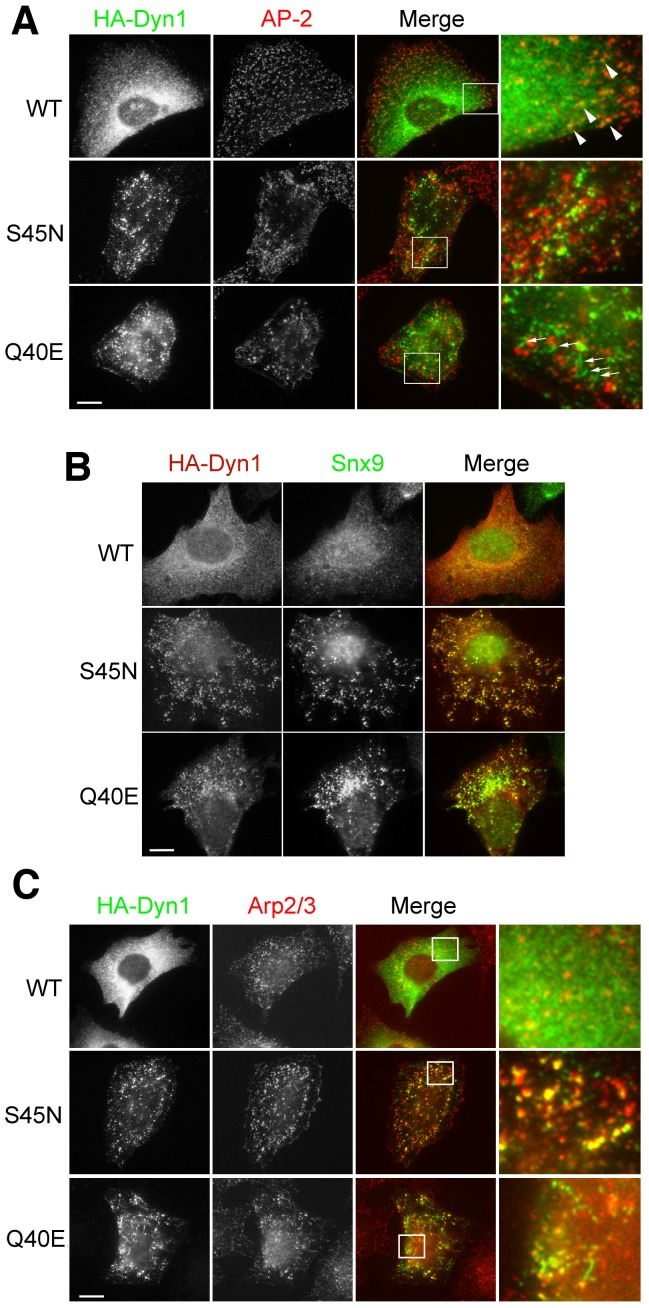
Accumulation of endocytic intermediates in cells expressing dominant-negative dynamin mutants. Indirect immunofluorescence of mouse fibroblasts acutely expressing the indicated HA-tagged dynamins, detected with anti-HA antibody (rabbit Y11, Santa Cruz or mouse 12CA5). (A) AP2 was detected with anti-α-adaptin Ab AP.6. (B), Snx9 was detected with anti-Snx9 Ab (Schmid lab) (C) Arp2/3 was detected with anti-Arp3 Ab (BD Transduction Lab). Scale bar, 10 µm. Representative cells from three independent experiments are shown.

Relative to WT dynamin-1, which was mainly dispersed in the cytosol, both mutants accumulated in clustered puncta at the plasma membrane (PM), which only partially co-localized with the clathrin adaptor protein AP-2 (Figure 2A, arrow head). However, AP-2 was often found just adjacent to mutant dynamins. The Q40E mutant could also be detected on tubular structures (Figure 2A, arrows), which were only rarely seen with S45N. In cells expressing mutant, but not WT dynamin-1, we also observed the redistribution of other endocytic accessory proteins (EAPs) from their disperse cytosolic staining to a more punctate pattern on the cell surface, substantially colocalizing with the mutant dynamins. These include SNX9 (Figure 2B), synaptojanin and intersectin (Figure S2B and C in File S1). These effects were indistinguishable in cells expressing either Q40E or S45N dynamin suggesting that these interactions are unlikely to require GTP binding. These findings are consistent with the fact that many of these partners interact with the proline/arginine-rich domain (PRD) of dynamin.

Dynamin has been suggested to control actin assembly. In Dyn1/2 double knock-out cells, massive rearrangements of the actin cytoskeleton occur and actin accumulates around trapped endocytic intermediates [38]. A more recent study pointed to a positive feedback loop between actin and dynamin recruitment to CCPs that was dependent on dynamin's GTPase activity [6]. We therefore examined actin and Arp2/3 localization in WT and mutant cells. Arp2/3 puncta detected in cells appeared larger/brighter in mutant cells and substantially colocalized with mutant dynamins (Figure 2C). Again, this redistribution appeared to be independent of GTP binding. Under these acute conditions, neither mutant altered the distribution of actin stress fibers (Figure S2D in File S1).

**Figure 3:**
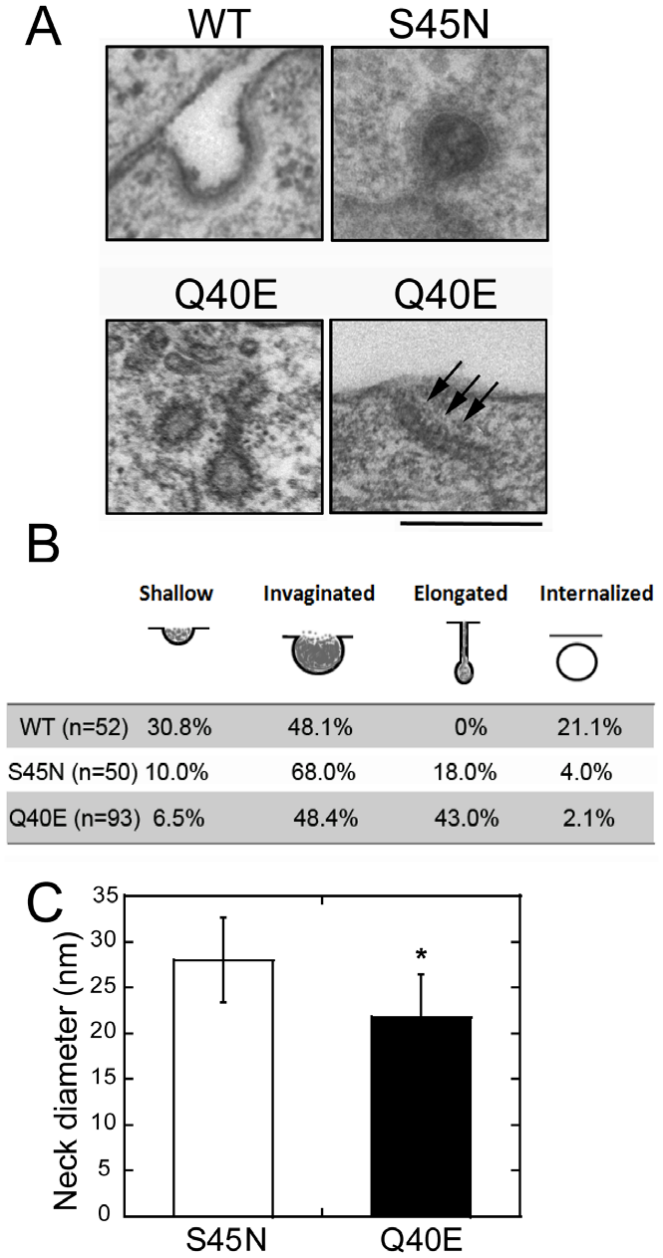
Ultrastructural analysis of clathrin coated pits (CCPs) accumulated in dynamin mutant cells. (A) Thin sections of tTA-HeLa cells acutely overexpressing the indicated dynamin-1 were prepared and imaged as described in *Experimental Procedures*. Scale bar, 250 nm. Representative cells from two independent experiments are shown. (B) Quantitation of CCP intermediates. Clathrin coated endocytic intermediates were counted and scored as shallow, invaginated, elongated or internalized structures. The results are expressed as % of total CCPs scored (n). (C) The average diameter of elongated necks (n = 30) detected in cells expressing S45N (28.0±4.7 nm) or Q40E (21.7±4.8 nm) was measured. The difference is significant, *p*<0.01 by Student *t-test*.

Previous studies detected ultrastructural differences in CCP intermediates that accumulate upon overexpression of different G domain mutants [9], [15], [35]. To observe the detailed structure of CCPs, we used ruthenium red labeling to determine whether the structures we observed remained connected to the cell surface. We measured 4 classes of clathrin-coated structures; shallow, invaginated, elongated (including detached coated structures that were ruthenium red positive) and internalized/ruthenium red-negative CCVs. As previously reported, invaginated CCPs that remained open to the cell surface predominantly accumulated in cells overexpressing S45N, although we also detected an increased number of elongated CCPs (Figure 3A and 3B). Intermediates accumulating in cells overexpressing Q40E were generally at later stages of CCP maturation and there was a pronounced accumulation of elongated CCPs relative to the S45N mutant. In many cases electron-dense bands around the elongated neck could be detected (Figure 3A, arrows), which were seldom seen in S45N-expressing cells. We also compared the diameter of the elongated necks detected in cells expressing Q40E or S45N (Figure 3C). The necks of elongated CCPs were on average more constricted in the Q40E expressing cells. Indeed the diameter of 21.7 nm is consistent with cryo-EM studies of GMPPCP-bound dynamin [39], [40]. Together, these data show that Q40E blocks at a later stage in CCP maturation than S45N.

### Dominant-negative effects on assembly-stimulated GTPase and membrane fission activities

Structural studies have shown that assembly-stimulated GTPase activity requires dimerization of G domains and that this dimerization occurs through longitudinal interactions between dynamin subunits on adjacent rungs of a spiral rather than through lateral interactions between adjacent subunits on the same rung [16]. Thus, the first active G domain dimer forms only after initiation of a second ring in an assembled dynamin collar. What is not known is whether GTP hydrolysis needs to be coordinated across the assembled dynamin collar for efficient fission or whether GTP hydrolysis by a single G domain dimer is sufficient to drive fission, i.e. what is the functional unit for membrane fission? We reasoned that a quantitative comparison of the dominant-negative effects of S45N, which is defective in GTP binding and Q40E, which, at 1 mM GTP can bind, but not hydrolyze GTP, could address these two questions.

**Figure 4:**
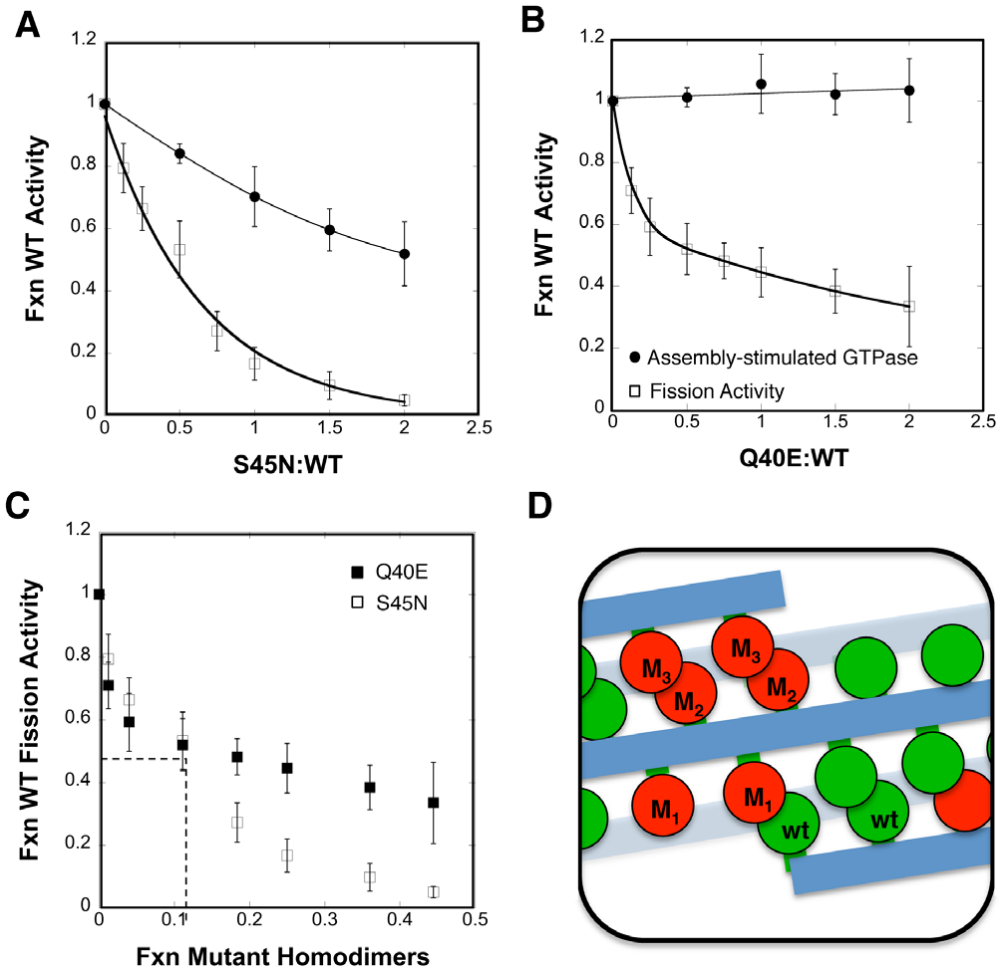
*In vitro* inhibitory effects of dominant-negative dynamin mutants. The inhibitory effects of increasing concentrations of S45N (A) and Q40E (B) mutants on the assembly-stimulated GTPase (•) and fission, i.e. the release of vesicles from SUPER templates (□) activities of 0.5 µM WT dynamin-1. The data (averages ± std. dev., n = 3 for GTPase assays, n = 11 for fission assays) are normalized to the activity of WT dynamin-1 alone and plotted as a function of the mutant∶WT ratio. Both assays were performed at room temperature in the presence of 1 mM GTP. (C) The inhibitory effects of the Q40E (▪) and S45N (□) mutants on the fission activity of WT dynamin-1, plotted as a function of the fraction of mutant dimers, assuming a binomial distribution. (D) Illustration of a dynamin collar formed by a 1∶1 mixture of mutant∶WT dynamin dimers. Under these conditions fission activity is inhibited ∼80% by S45N mutant and ∼60% by the Q40E mutant.

For these experiments we measured the liposome-stimulated GTPase and fission activities (i.e. the ability to release vesicles from SUPER templates) of 0.5 µM WT in the presence of increasing amounts of S45N or Q40E mutant dynamin-1. GTPase assays were performed at saturating levels of lipid (150 µM) and 1 mM GTP to ensure that neither substrate was limiting, and at room temperature to parallel conditions for the membrane fission assay. S45N, which cannot bind GTP, was also unable to fully activate its partner subunit and thus inhibited the assembly-stimulated GTPase activity of WT dynamin (Figure 4A, closed circles). At equimolar WT∶S45N, WT G domains have an equal probability of forming homodimers with WT partners or heterodimers with S45N partners. Under these conditions the total assembly-stimulated GTPase activity of WT G domains was reduced by 30%. Similar data obtained at different WT∶S45N ratios allowed us to calculate that WT G domains in WT∶S45N heterodimers exhibited <40% the activity of G domains present in WT homodimers (see Figure S3 in File S1). Thus, while not essential, GTP binding is necessary for full activation of the partner subunit in the functional dimer. In contrast, Q40E had no effect on the assembly-stimulated GTPase activity of WT dynamin even at two-fold excess (Figure 4B, closed circles). From this we conclude that Q40E, despite its own severe defect in assembly-stimulated GTP hydrolysis, is fully capable of forming a functional heterodimer and stimulating the GTPase activity of its WT partner.

In contrast to GTPase activity, fission activity was highly sensitive to addition of even small amounts of either S45N or Q40E mutants, such that ∼50% inhibition was observed at a mutant∶WT ratio of 0.5 (Figure 4A and B, open squares). Given that WT G domains in Q40E∶WT heterodimers are fully active for assembly-stimulated GTPase activity, we plotted these data as a function of the fraction of mutant homodimers/total (Fig. 4C). Strikingly, the presence of only ∼1 mutant homodimer in ten resulted in a 50% inhibition of fission activity. The differential sensitivity of WT dynamin's assembly-stimulated GTPase and fission activities to the dominant-negative effect of the mutants suggests that while the functional unit for the former is a G domain dimer, the functional unit for dynamin-catalyzed fission is much larger. Given that active G domain dimers form only at the interface between rings, and ∼12 dynamin G domains extend from each rung towards the interface [40], then two full rings would be required to generate the 10–12 G domain dimers (diagrammed in Figure 4D) required for maximum fission activity. Our data shows that as little as two inactive G domain dimers per double ring would severely impair fission activity. Thus, we conclude that coordinated GTP hydrolysis throughout the ring is necessary to efficiently drive membrane fission. Interestingly, inhibition by the Q40E mutant reaches a plateau relative to that caused by S45N (Figure 4B and 4C). This likely reflects the half-site activity of Q40E∶WT heterodimers relative to the lower activity of S45N∶WT heterodimers, and thus the ability of these assemblies to more readily undergo cycles of GTP hydrolysis and disassembly.

## Discussion

Our analyses of two distinct classes of dynamin GTPase mutants helps to resolve the different roles of GTP binding, basal and assembly-stimulated GTP hydrolysis in dynamin function during clathrin-mediated endocytosis and for catalyzing membrane fission. There has been controversy as to both the magnitude and physiological relevance of dynamin's basal rate of GTP hydrolysis [10], [15]. Although the minimal, monomeric GG exhibits the same basal GTPase activity as full length dynamin [36], there also remains uncertainty as to whether or not dynamin's basal activity requires G domain dimerization [10]. The enzymatic properties of the Q40E mutation, characterized in more detail here, strongly argue that basal GTPase activity is independent of G domain dimerization.

A major finding of these studies is that, compared to assembly-stimulated GTPase activity resulting from dimerization of G domains, fission activity of a fixed number of WT dynamin molecules is more sensitive to inhibition by both S45N and Q40E mutants. This is particularly apparent for Q40E which does not diminish the assembly-stimulated GTPase activity of WT dynamin upon co-polymerization on a membrane template, yet robustly inhibits the ability of WT dynamin to catalyze membrane fission. The above result has two important corollaries. First, the higher sensitivity to the dominant-negative effects of the mutants reveals that the minimal functional unit supporting dynamin-catalyzed membrane fission is considerably larger than that for assembly-stimulated GTPase activity. For S45N, the IC50 for inhibition of GTPase activity is 5-fold greater than for inhibition of fission activity, suggesting that the functional unit for fission consists of ∼10 subunits rather than 2. Given that G domain dimerization occurs between subunits on adjacent rungs, these data suggest that the function unit for fission involves ∼2 rungs (12 subunits/rung). Second, inhibition of fission by Q40E, demonstrates that both G domains of dimers must undergo coordinated GTP hydrolysis. From these data we conclude that the minimum apparatus for dynamin-catalyzed fission on SUPER templates must span the length of ∼two rungs of a dynamin spiral and that coordinated GTP hydrolysis throughout this assembly is required for dynamin alone to efficiently catalyze fission. Further studies are needed to determine the functional consequences of this coordinated GTPase activity. Does it drive a concerted conformational change, such as an abrupt constriction of the collar, coordinated twisting or induction of stress in the underlying membrane, or instead is it required for rapid synchronous disassembly of the collar? It will also be important to determine what and how other cellular factors, such as epsin [31] and BAR domain-containing proteins [38] affect the efficiency and mechanism of dynamin-catalyzed membrane fission both *in vitro* and *in vivo*.

In addition to the above, we have shown that acute overexpression of Q40E dynamin, which is most severely impaired in assembly-stimulated GTPase activity, blocks CME at a later stage than S45N, which is severely impaired in both GTP binding and basal GTPase activities. These data are consistent with several studies suggesting that dynamin's basal GTP binding and/or hydrolysis activities play additional, yet still poorly defined, roles in early stages of CCV formation [9], [33], [35] whereas dynamin self-assembly and assembly-stimulated GTPase activities are required for membrane fission at the terminal stages of CCV formation. Long-necked endocytic intermediates have been observed in other studies in which dominant-negative dynamin mutants (e.g. K44A, T65A) are overexpressed [15], [35]. These studies involved much longer-term overexpression (16–20 hr) than the acute overexpression (3 h) protocol used here. These shorter incubation times may have enabled us to detect greater differences in the intermediates that accumulate. Indeed, omega-shaped, collared structures accumulate at the synapse of *shibire* flies, which express a temperature-sensitive mutant of dynamin, after brief incubation at the nonpermissive temperature. These structures develop long necks only after subsequent incubation [41].

Recent time-lapse microscopic analyses of cells overexpressing a series of hypomorphic GTPase domain mutants, have also suggested that dynamin functions prior to self-assembly at early stages of CME, in part to regulate actin dynamics at CCPs [6]. Based on differential effects seen in cells expressing a series of biochemically distinct, hypomorphic G domain mutants, these authors concluded that the GTPase cycle of dynamin regulates the rate of recruitment of both actin and the dynamin-binding partner, endophilin 2. Although we did not measure recruitment kinetics, we were unable to detect differences in the steady-state level of recruitment of endocytic accessory factors or the actin regulatory Arp2/3 complex to CCP intermediates stalled in cells expressing either S45N or Q40E dynamin. These proteins are likely recruited to CCPs either through nucleotide-independent interactions with dynamin's PRD or through direct or indirect interactions with other components of the clathrin coat. Despite the increased localization of Arp2/3 to stalled CCPs in cells overexpressing S45N or Q40E mutants, we did not detect changes in the overall distribution of actin filaments. Our results differ from the dramatic redistribution of actin fibers seen in Dyn1/2 knock-out cells [38]. These differences may be due to indirect effects and/or compensatory responses associated with the long (5 day) period of dynamin depletion as compared to our acute (1 h) induction period. Indeed, cdc42 signaling was shown to be constitutively activated and required for the actin accumulation seen in Dyn1/2 null cells [42]. Alternatively, if dynamin functions to regulate actin assembly at sites of endocytosis, it must do so in a manner independent of its ability to bind GTP.

In summary, utilizing two distinct classes of dynamin GTPase mutants, we demonstrate sequential roles for dynamin's GTP binding and assembly-stimulated GTPase activity during CME in cultured cells. We have also demonstrated that coordinated GTP hydrolysis, presumably leading to a concerted conformational change throughout the assembled dynamin collar are required for fission. The nature of this conformational change and its consequences on the global structure of assembled dynamin remains to be determined.

## Materials and Methods

### Dynamin purification and GTPase activity

Wild type and mutant dynamins were expressed by transient transfection in Sf9 cells and purified by affinity chromatography using GST-tagged amphiphysin-II SH3 domain as described previously [22]. Basal and liposome-stimulated GTP hydrolysis by dynamin was measured as a function of time using a colorimetric malachite green assay that detects the release of inorganic phosphate [43]. Liposomes (DOPC∶DOPS∶PIP2 at 80∶15∶5) were prepared by extrusion through polycarbonate membranes (Whatman, Clifton, NJ) with a pore size of 0.1 µm using an Avanti Mini-Extruder. The stimulated-GTPase activity measured in this work was performed at room temperature, the same temperature used for fission assays.

The Michaelis constant, *K_m_*, for dynamin GTPase activity was obtained through fitting the GTP hydrolysis rate vs. GTP concentration into the Michaelis-Menton equation (V = *k_cat_* [E][S]/*K_m_*+[S]). *K_m_* is also equal to (k*_off_*+*k_cat_*)/k*_on_*. Therefore, when k*_off_*≫*k_cat_*, *K_m_* approaches k*_off_*/k*_on_* = *K_d_*.

### Membrane fission and curvature generation

Membrane fission and curvature generation abilities of dynamin were analyzed as described before with SUPER templates [24], [25]. Briefly, 5×10^5^ SUPER templates were added to 100 µL buffer containing 20 mM Hepes (pH 7.5), 150 mM KCl, 1 mM MgCl_2_, 1 mM GTP, and 0.5 µM dynamin for 30 min at room temperature. Templates were pelleted and the supernatant was mixed with Triton X-100 to dissolve vesicles. Total fluorescence of templates was determined in a separate reaction containing 5×10^5^ SUPER templates in Triton X-100. Fluorescence was measured, and fission activity is expressed as the percentage of total fluorescence on SUPER templates. For curvature generation analysis, the SUPER templates were incubated with dynamin in the absence of GTP and observed under fluorescent microscope (inverted Olympus IX-70 microscope using a 100×, 1.35NA oil immersion objective), as previously described [22].

### Acute expression of dynamin and Tfn uptake assays

For high infection efficiency and acute expression of exogenous dynamin, tTA-HeLa cells expressing the chimeric tet-regulatable transcription activator were infected with adenovirus for 2 hours in the presence of tetracycline to inhibit transcription. After washing, the infected tTA-HeLa cells were incubated with tetracycline-free medium containing cycloheximide (10 µg/ml) to accumulate dynamin-encoding mRNA for 3 hours [44]. Acute expression of dynamin was then induced for 1 hour by removal of cycloheximide. B-Tfn internalization was performed as described, using B-Tfn as a ligand and assessing its internalization into an avidin- or MesNa-inaccessible compartment [35].

### Immunofluorescence staining

We used mouse fibroblast cells [45] for indirect immunofluorescence stain because of their better morphology. Thus, fibroblasts acutely expressing dynamin, as described above, were fixed and permeabilized simultaneously with 2% warm paraformaldehyde and 0.5% TX-100 for 2 min to reduce cytosolic dynamin signals [45] and then further fixed with 4% paraformaldehyde for 40 min. After blocking with 2% BSA, cells were stained with the indicated primary and secondary antibodies. Cells were observed under wide-field epifluorescence microscopy using an inverted Olympus IX-70 microscope with a 100×, 1.35 numerical aperture (NA) oil-immersion objective.

### Electron Microscopy

tTA-HeLa cells acutely overexpressing either Dyn1 WT or mutants were fixed in the presence of ruthenium red, embedded in epon and prepared for thin-section EM as previously described [35]. Briefly, the cells were fixed for 1 h with 1.2% glutaraldehyde containing 0.5 mg/ml ruthenium red at room temperature, and unstained ultrathin sections were observed under an electron microscope. At least 50 clathrin-coated structures in each sample were counted and categorized into one of 4 stages as illustrated in Figure 3B.

## Supporting Information

File S1 **A pdf containing the following three Supplemental Figures and their legends:** Figure S1 showing GTPase activity of WT and Q40G minimal GTPase-GED fusion proteins expressed and purified from E. Coli; Figure S2 showing transient expression of WT and mutant dynamins in tTA-HeLa cells and their effects on subcellular distribution of intersectin, synaptojanin and actin; Figure S3 showing a plot of the fraction of WT G domains vs S45N∶WT ratio assuming a binomial distribution and a fit of assembly-stimulated GTPase data demonstrating that WT GTPase activity of WT∶S45N heterodimer is 35% that of WT homodimers.
(PDF)
