# Supplementary material for: Dynamin-Catalyzed Membrane Fission Requires Coordinated GTP Hydrolysis
Source: PLoS One. 2013 Jan 31;8(1):e55691. doi: 10.1371/journal.pone.0055691 (PMC3561337; doi:10.1371/journal.pone.0055691)
Supplement: File S1 — A pdf containing the following three Supplemental Figures and their legends: Figure S1 showing GTPase activity of WT and Q40G minimal GTPase-GED fusion proteins expressed and purified from E. Coli; Figure S2 showing transient expression of WT and mutant dynamins in tTA-HeLa cells and their effects on subcellular distribution of intersectin, synaptojanin and actin; Figure S3 showing a plot of the fraction of WT G domains vs S45N∶WT ratio assuming a binomial distribution and a fit of assembly-stimulated GTPase data demonstrating that WT GTPase activity of WT∶S45N heterodimer is 35% that of WT homodimers. (PDF) [file pone.0055691.s001.pdf]

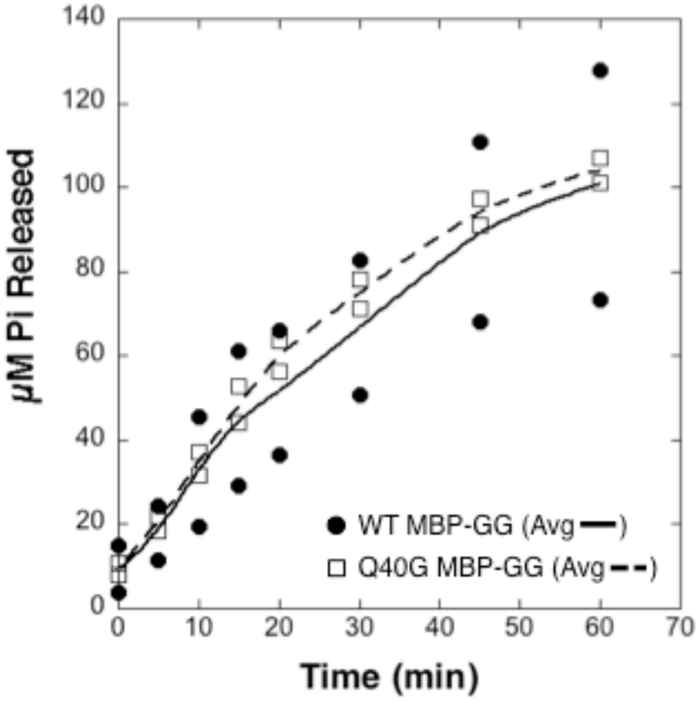

Supplemental Figure 1. GTPase activity of WT and Q40G minimal GTPase-GED fusion proteins. Shown are two independent GTPase assays of E. Coli expressed wild-type MBP-GG (●) or Q40G mutant MBP-GG (❑) measured in the presence of 1 mM GTP exactly as previously described (Chappie et al., 2010). Lines correspond to the average of the two experiments.

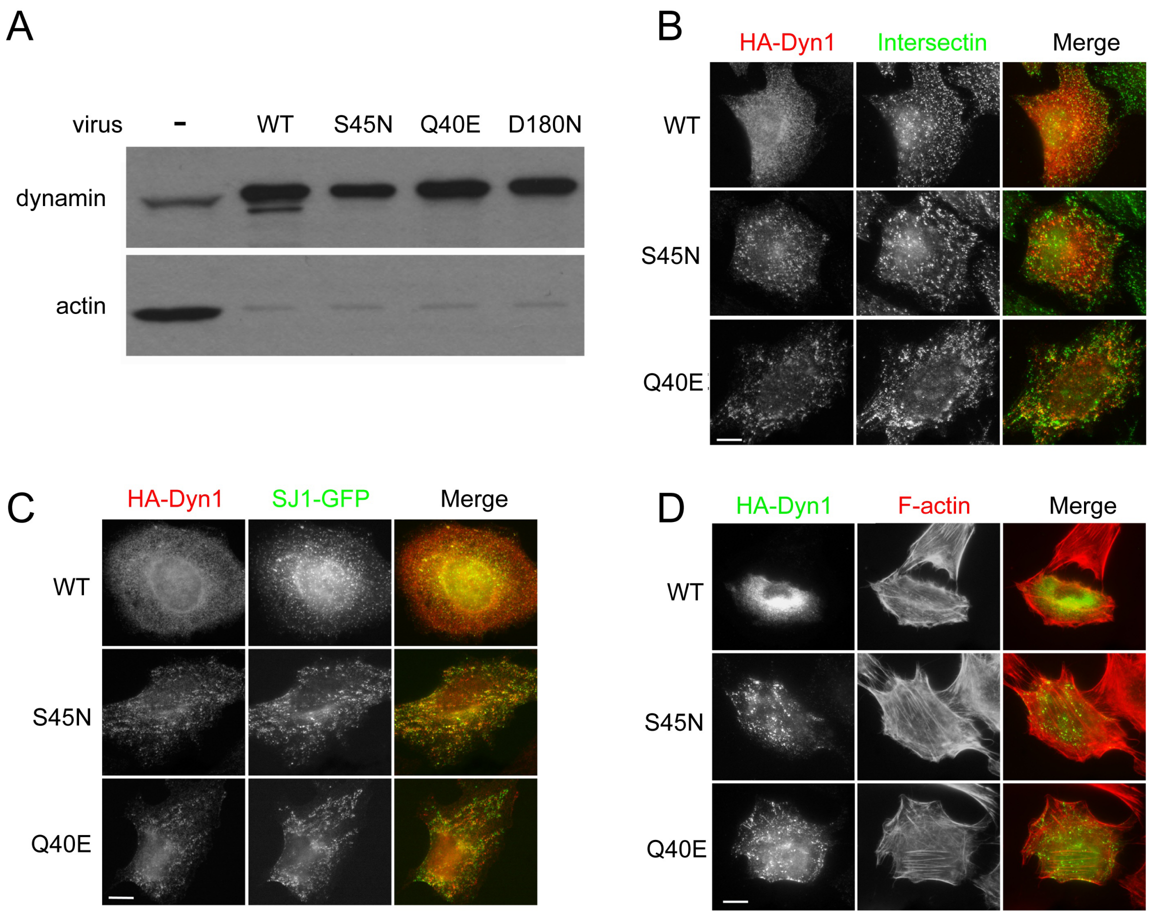

Liu et al. Supplemental Figure 2

Supplemental Figure 2, Expression and effects of dynamin mutants. (A) Acute expression of exogenous dynamins. Adenoviral-mediated dynamin expression was quantified with anti-dynamin (Schmid lab, #2960) and anti-actin (MAB1501R, Chemicon International, Temecula, CA) antibodies. Ten-fold less extract from infected cells was loaded onto the gel to have comparable signals of dynamin. (B, C and D) Effects of dynamin overexpression on the distribution of endocytic accessory proteins or actin stress fibers. Mouse fibroblast cells acutely expressing the indicated dynamins were stained with antibodies against intersectin (Novus Biologicals, Littleton, CO) (B), with phalloidin (Invitrogen) (D) or co-expressing synaptojanin1-GFP (C).

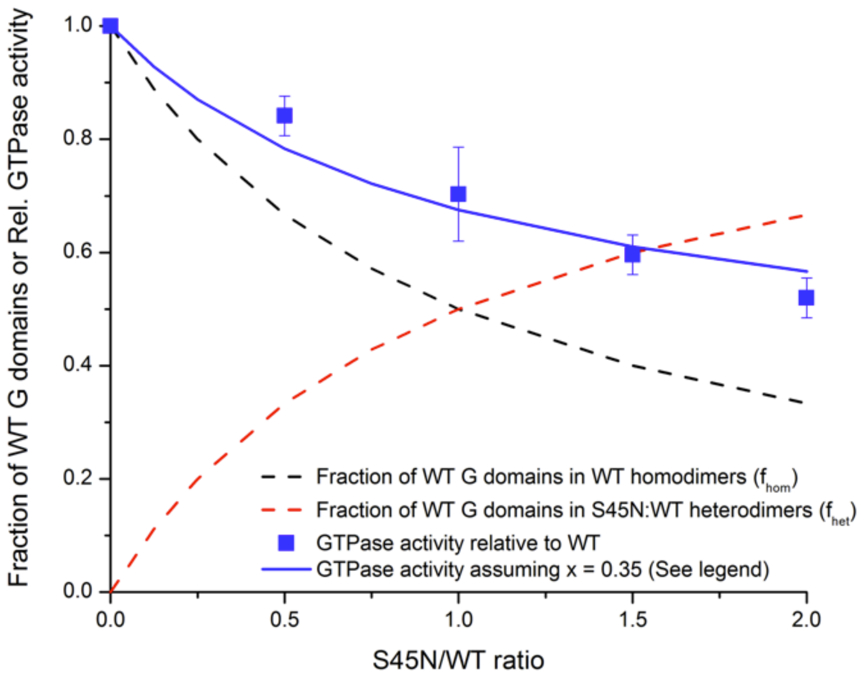

Supplemental Figure 3. GTP binding by both subunits of a GG dimer is required for full GTPase activity. The fraction of WT G domains in WT homodimers and S45N:WT heterodimers (f_{hom} and f_{het}, respectively) was calculated assuming that the probability distribution of WT and mutant homodimers and mutant:WT heterodimers formed upon assembly on a membrane template is dependent only the relative abundances of the two dynamin species and is thus binomial. Since the GTPase activity in each tested condition originates from a fixed amount of WT dynamin G domains, the total GTPase activity in mutant:WT mixtures relative to pure WT = $f_{hom} + x \times f_{het}$, where x is the specific activity of a WT G domain in a heterodimer relative to WT G domain in a homodimer. By systemically varying x, it was found that best visual fit to the data was achieved with x = 0.35, indicating that the WT G domains in S45N:WT heterodimers were only ~35% as efficient in hydrolyzing GTP as those in WT homodimers.
